# Evaluating a Human Ear-Inspired Sound Pressure Amplification Structure with Fabry–Perot Acoustic Sensor Using Graphene Diaphragm

**DOI:** 10.3390/nano11092284

**Published:** 2021-09-02

**Authors:** Cheng Li, Xi Xiao, Yang Liu, Xuefeng Song

**Affiliations:** 1 School of Instrumentation Science and Opto-Electronics Engineering, Beihang University, Beijing 100191, China; xiaoxi@buaa.edu.cn (X.X.); sy2017328@buaa.edu.cn (Y.L.); 2 Research Institute of Beihang University in Shenzhen, Shenzhen 518055, China; 3 Shenzhen Institute for Quantum Science and Engineering, Southern University of Science and Technology, Shenzhen 518055, China; song@sustc.edu.cn

**Keywords:** sound pressure amplification structure, double diaphragm scheme, Fabry–Perot sensor, graphene diaphragm, sensitivity enhancement

## Abstract

In order to enhance the sensitivity of a Fabry–Perot (F-P) acoustic sensor without the need of fabricating complicated structures of the acoustic-sensitive diaphragm, a mini-type external sound pressure amplification structure (SPAS) with double 10 μm thickness E-shaped diaphragms of different sizes interconnected with a 5 mm length tapered circular rod was developed based on the acoustic sensitive mechanism of the ossicular chain in the human middle ear. The influence of thickness and Young’s modulus of the two diaphragms with the diameters of 15 mm and 3 mm, respectively, on the amplification ratio and frequency response were investigated via COMSOL acoustic field simulation, thereby confirming the dominated effect. Then, three kinds of dual-diaphragm schemes relating to steel and thermoplastic polyurethanes (TPU) materials were introduced to fabricate the corresponding SPASs. The acoustic test showed that the first scheme achieved a high resonant response frequency with lower acoustic amplification due to strong equivalent stiffness; in contrast, the second scheme offered a high acoustic amplification but reduced frequency range. As a result of sensitivity enhancement, adapted with the steel/TPU diaphragm structure, an optical fiber Fabry–Perot sensor using a multilayer graphene diaphragm with a diameter of 125 μm demonstrated a remarkable sensitivity of 565.3 mV/Pa @1.2 kHz due to the amplification ratio of up to ~29.9 in the range of 0.2–2.3 kHz, which can be further improved by miniaturizing structure dimension, along with the use of microstructure packaging technology.

## 1. Introduction

Acoustic pressure sensing plays an important role in applications such as environmental noise monitoring [1], photoacoustic spectroscopy [2] and human–machine interaction [3], etc. Due to the advantages including immunity to electromagnetic interference, the capability of performing remote sensing, very high resolution, fast response, and compact size [4], optical sensors have been attracting more research interests among various acoustic sensors. It is important to note that planar diaphragms are generally used to sense the sound pressure in optical sensors. To strengthen acoustic sensitivity, various diaphragm structures have been reported in recent years. The diaphragms with large areas and small thickness were generally adopted to increase the sensitivity [5]. Unfortunately, a larger ratio of diameter to thickness may lead to low resonance frequency and uneven frequency response [6,7,8]. Although a metal diaphragm-based sensor developed by Gaomi Wu [9] showed a sensitivity larger than 800 mV/Pa, the high sensitivity mainly resulted from the larger film area. When the film diameter was reduced to 125 μm, the diaphragm with the same area ratio would only have an extremely low sensitivity of 8 mV/Pa. Recently, the corrugated diaphragm was introduced to improve acoustic sensitivity [10,11,12,13,14]. For example, the average mechanical sensitivity of the corrugated silver diaphragm-based optical fiber microphone was 52 nm/Pa in the low frequency range of 63 Hz–1 kHz, which was twice that of the planar diaphragm with the same larger diameter of 2.5 mm and thickness of 200 nm [13]. In their latest work in 2020 [14], the mechanical sensitivity was increased to 82.65 nm/Pa by optimizing the depth of the corrugated diaphragm. However, fabricating a corrugated diaphragm is limited by the diaphragm material and complicated micro-nano fabrication technology, in addition to the need for a larger size diaphragm. Instead of the use of a peripherally clamped circular diaphragm, cantilever type sensors have been developed [15,16,17,18]. In Ref. [17], the mechanical sensitivity of 198.3 nm/Pa at 1 kHz was demonstrated by using a steel cantilever with the larger size of 1.8 mm × 1 mm and the thickness of 10 μm. Although the cantilever structure has the advantage of high sensitivity, the asymmetric feature makes the sensor mainly operate at close to the resonance frequency with an extremely narrow band and nonlinear response [19]. Another feasible way is to employ external acoustic amplification structures. In 2011, Eui Sung Jung [20] proposed a microphone with a spiral-type acoustic tube, which generated a resonance effect between the diaphragm and the acoustic transducer inside a case. The underwater frequency response of the microphone at 3–4 kHz was improved by approximately 20 dB. Nevertheless, this structure was specifically for sensing diaphragms with large areas, especially those with a diameter of over 10 mm. Then, in 2015, Pan Hu [21] designed a steel megaphone at the front of an Fabry–Perot (F-P) acoustic sensor, which showed the sensitivity of 56.99 mV/Pa at 4.5 kHz. However, the long megaphone was confined to short-distance sound detection. Recently, in 2018, Renxi Gao [22] proposed a Helmholtz resonator to encapsulate an optical fiber vibration sensor with multiple response peaks below 1 kHz; however, the bandwidth of each resonance peak was less than 100 Hz, meaning an extremely uneven frequency response. Therefore, an external sound pressure-enhanced structure suitable for miniaturized sensors, capable of long-distance detection in a wide frequency range, is of great significance to F-P acoustic sensors.

In reality, human organs have subtle sound sensing structures that have recently been used to improve the frequency response and sensitivity. As we know, the basilar membrane (BM) in the human cochlear is naturally designed with an asymmetric trapezoidal shape that has numerous resonance frequencies by varying the width and thickness of the BM along the cochlea spiral, thereby enabling the resonance-based sensing and frequency tuning capability [23]. For example, in 2016, Jongmoon Jang [24] reported a trapezoidal-shape triboelectric-based artificial basilar membrane (TEABM) using eight double-ended polymer beams fixed on the substrate. The beam length was in the range of 8.2–32 mm and its width was 6–8 mm. Compared with their previous artificial basement membrane with a cantilever array, this fabricated TEABM displayed multiple harmonic response spikes in the lower range of 294.8–2311 Hz, and the sensitivity to sound was improved from 0.35–1.67 mV/Pa to 1.74–13.1 mV/Pa. Then, in 2018, Jae Hyun Han [23] developed a curved-shape basilar membrane-inspired flexible piezoelectric acoustic sensor (f-PAS) using seven polymer beams with a length in the range of 0.5–1 mm on the plastic substrate. The curved-shape f-PAS exhibited a non-flat resonance response distribution composed of many discrete harmonic peaks in the range of 0.1–4 kHz. The acoustic test showed four to eight times higher sensitivity compared to the conventional condenser sensor. However, multi-channel simultaneous detection is also required for the beam array structure due to the limited effective bandwidth, therefore restricting the miniaturization and complicating the potential application because of the access of multiple acoustic probes and their matching conditioning devices. Furthermore, biological studies on the human ear have verified that external sound pressure is transmitted and then amplified from the tympanic membrane to the stapes footplate in the middle ear [25,26,27], wherein the middle-ear sound pressure gain can reach 23.5 dB around 1–2 kHz. As a result, the middle ear-inspired sound pressure amplification structure (SPAS) is a great option for enhancing acoustic sensitivity.

Hence, in this paper, a simple low-frequency SPAS primarily consisting of dual diaphragms of different diameters interconnected with a tapered rod was introduced to drive the adapting F-P acoustic sensor by imposing the amplified sound pressure onto the graphene diaphragm adhered on the endface of the sensor, which offers a feasible sensitivity-enhanced way without the need of modifying an F-P sensor by fabricating complicated diaphragm structures. Then, in virtue of the sound reinforcement principle of the human middle ear, the acoustic amplification performance of the developed structure was investigated for the determination of the structural parameters of SPAS by COMSOL Multiphysics simulation. Then, three types of SPASs were fabricated and further evaluated by acoustic test, which characterized the significant role of acoustic amplification for SPAS and a high acoustic sensitivity of 255.5 mV/Pa @ 1 kHz was achieved for an F-P acoustic sensor with SPAS. Compared with the external amplification structures previously reported in Refs. [20,22], the simple bionic SPAS can offer an enhanced sensitivity in a relatively wide frequency band ranging from 0.2 Hz to 2.3 kHz. It is necessary to note that, herein, the F-P acoustic sensor adapted with the SPAS employed a graphene diaphragm due to the small thickness and excellent mechanical strength of graphene [28]. Although recent research described a large-area graphene oxide (GO) diaphragm-based F-P sensor with a sensitivity of 25.84 mV/Pa [29], it is more appropriate for the SPAS to use a smaller cavity accommodating the F-P probe. Hence, the F-P acoustic sensor with graphene diaphragm was introduced in the manuscript to evaluate the SPAS performance.

## 2. The Model Adapted to SPAS

**Figure 1:**
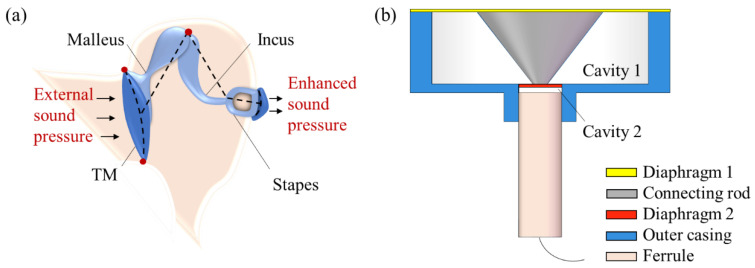
Schematic illustrations of (**a**) a human middle ear structure and (**b**) the presented SPAS with an F-P probe.

Figure 1 illustrates the schematic diagram of the SPAS. Since the lymphatic fluid that is full of the human cochlea would cause higher acoustic loss, an ossicular chain structure in the middle ear is capable of enhancing the input external acoustic pressure and then transfer into the inner ear for effectively matching sound passing from the low impedance of external air to the high impedance of cochlear fluid [25]. It is known that a human middle ear is made of a tympanic membrane ™, middle ear cavity, and ossicular chain which consists of the malleus, incus, and stapes [30]. Referring to the structural illustration of a human middle ear in Figure 1a, external air pressure is strengthened via large TM vibration and then lever amplification offered by malleus and incus. In this case, the amplified acoustic pressure is passed to the following inner ear by the use of the stapes. According to the acoustic signal enhancement scheme including the large area ratio between input TM and output stapes and the lever magnifying mechanism formed by malleus and incus, a mini-type SPAS of simple structure with a large area ratio of the outer film (diaphragm 1) to inner film (diaphragm 2) is proposed, as shown in Figure 1b, wherein the two films of different sizes are equivalent as the TM and stapes in Figure 1a for acoustic signal amplification. Then, a conical rod, serving as a pressure transmission medium, is fixedly connected with the two diaphragms at its upper and lower endfaces, respectively. The diaphragms attached with the rod have adhered to the supporting outer casing that also offers a guide tube for assembling an F-P probe, thereby forming two sealed air cavities (cavity 1 and cavity 2). The compressed air in cavity 2 caused by the SPAS would increase the pressure imposed on the sensitive diaphragm such as graphene suspended on the ferrule endface. As a consequence, acoustic sensitivity can be improved with the aid of the SPAS for a diaphragm-typed F-P acoustic sensor.

**Figure 2:**
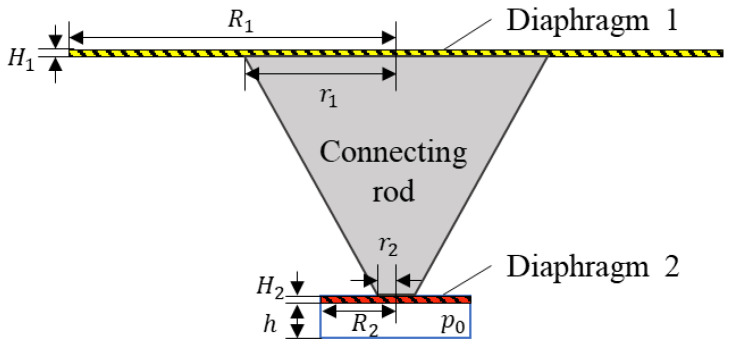
An equivalent schematic diagram of the force model for the presented SPAS.

For the SPAS displayed in Figure 1b, an equivalent schematic diagram of the force model is depicted in Figure 2. For the sensitivity increase, the radius *R*_1_ of the diaphragm 1 is five times larger than the radius *R*_2_ of the diaphragm 2. In this case, considering the smaller deflection deformation of diaphragm 1 resulted from the applied lower external acoustic pressure ranging from 20 μPa to 20 Pa, the relative volume change in cavity 1 is by far lower than that in cavity 2, thus neglecting the effect of pressure change in cavity 1. For the purpose of modeling the load-deflection behavior, in view of the rigid connecting rod, the effective working part of each diaphragm is an annular region, which can be regarded as an E-shaped diaphragm under the uniform load on one side and the concentrated load on the other side. According to the small deformation theory of the E-shaped plates [31], the normal displacement *ω*_1_(*r*) about the distance *r* from the center of diaphragm 1 under the uniform dynamic load *p*_1_ and the central load *p_c_*_1_ can be approximated as:

(1) $$\left\{ \begin{array}{l} \omega_{1}\left(r\right)=\frac{12\left(1-\mu_{1}{}^{2}\right)}{E_{1}H_{1}{}^{3}}[\frac{r^{4}}{64}p_{1}-\frac{p_{c1}{r_{1}}^{2}r^{2}}{8}\left(\ln\frac{r}{R_{1}}-1\right)\\ \;\;\;\;\;\;\;\;\;\;\;\;\;+\frac{C_{1}}{4}r^{2}+C_{2}\ln\frac{r}{R_{1}}+C_{3}]\\ C_{1}=-\frac{p_{c1}{r_{1}}^{4}\ln\frac{r_{1}}{R_{1}}}{2\left({R_{1}}^{2}-{r_{1}}^{2}\right)}-\frac{p_{c1}{r_{1}}^{2}}{4}-\frac{p_{1}}{8}\left({R_{1}}^{2}+{r_{1}}^{2}\right)\\ C_{2}=\frac{p_{1}}{16}{R_{1}}^{2}{r_{1}}^{2}+\frac{p_{c1}{R_{1}}^{2}{r_{1}}^{4}}{4\left({R_{1}}^{2}-{r_{1}}^{2}\right)}\ln\frac{r_{1}}{R_{1}}\\ C_{3}=\frac{p_{1}}{64}{R_{1}}^{2}\left({R_{1}}^{2}+2{r_{1}}^{2}\right)+\frac{p_{c1}{R_{1}}^{2}{r_{1}}^{4}}{8\left({R_{1}}^{2}-{r_{1}}^{2}\right)}\ln\frac{r_{1}}{R_{1}}-\frac{p_{c1}{R_{1}}^{2}{r_{1}}^{2}}{16} \end{array}\right.$$

where *R*_1_ and *H*_1_ are the radius and the thickness of diaphragm 1, respectively; *r*_1_ is the radius of the upper endface of the connecting rod; *E*_1_ and *μ*_1_ are Young’s modulus and Poisson’s ratio of the diaphragm material. Similarly, the deflection *ω*_2_(*r*) of diaphragm 2 under the uniform dynamic load *p*_2_ and the central load *p_c_*_2_ can be obtained by:

(2) $$\left\{ \begin{array}{l} \omega_{2}\left(r\right)=-\frac{12\left(1-\mu_{2}{}^{2}\right)}{E_{2}H_{2}{}^{3}}[\frac{r^{4}}{64}p_{2}-\frac{p_{c2}{r_{2}}^{2}r^{2}}{8}\left(\ln\frac{r}{R_{2}}-1\right)\\ \;\;\;\;\;\;\;\;\;\;\;\;\;+\frac{C_{4}}{4}r^{2}+C_{5}\ln\frac{r}{R_{2}}+C_{6}]\\ C_{4}=-\frac{p_{c2}{r_{2}}^{4}\ln\frac{r_{2}}{R_{2}}}{2\left({R_{2}}^{2}-{r_{2}}^{2}\right)}-\frac{p_{c2}{r_{2}}^{2}}{4}-\frac{p_{2}}{8}\left({R_{2}}^{2}+{r_{2}}^{2}\right)\\ C_{5}=\frac{p_{2}}{16}{R_{2}}^{2}{r_{2}}^{2}+\frac{p_{c2}{R_{2}}^{2}{r_{2}}^{4}}{4\left({R_{2}}^{2}-{r_{2}}^{2}\right)}\ln\frac{r_{2}}{R_{2}}\\ C_{6}=\frac{p_{2}}{64}{R_{2}}^{2}\left({R_{2}}^{2}+2{r_{2}}^{2}\right)+\frac{p_{c2}{R_{2}}^{2}{r_{2}}^{4}}{8\left({R_{2}}^{2}-{r_{2}}^{2}\right)}\ln\frac{r_{2}}{R_{2}}-\frac{p_{c2}{R_{2}}^{2}{r_{2}}^{2}}{16} \end{array}\right.$$

where *R*_2_ and *H*_2_ are the radius and the thickness of diaphragm 1, respectively; *r*_2_ is the radius of the lower endface of the connecting rod; *E*_2_ and *μ*_2_ are Young’s modulus and Poisson’s ratio of the diaphragm material.

Due to the rigid connection of the tapered rod with the diaphragms 1 and 2, the center deflection for the two diaphragms can be approximated as:

(3) $$\omega_{1}(r_{1})=\omega_{2}(r_{2})$$

Then, based on the force conduction in the rod, the following relationship can be obtained by:

(4) $$\pi {r_{1}}^{2}p_{c1}=\pi {r_{2}}^{2}p_{c2}$$

In terms of the ideal gas equation of state [32], the air pressure (*p*_0_ + *p*_2_) in cavity 2 at a constant room temperature can be given by:

(5) $$p_{0}+p_{2}=\frac{\pi {R_{2}}^{2}hp_{0}}{\pi {R_{2}}^{2}h-\Delta V}$$

where *p*_0_ and ∆*V* are the initial pressure (1 × 10^5^ Pa) and the variation of volume in the cavity 2, respectively; *h* is the height of cavity 2. ∆*V* can be determined by:

(6) $$\Delta V=\pi {r_{2}}^{2}\omega_{2}(r_{2})+\int_{r_{2}}^{R_{2}}2\pi r\omega_{2}(r)dr$$

In this case, the generated dynamic acoustic load *p*_2_ in cavity 2 can be further written by:

(7) $$p_{2}=\left[\frac{\pi {R_{2}}^{2}h}{\pi {R_{2}}^{2}h-\left(\pi {r_{2}}^{2}\omega_{2}(r_{2})+\int_{r_{2}}^{R_{2}}2\pi r\omega_{2}(r)dr\right)}-1\right]p_{0}$$

For a specific SPAS with known structural parameters, due to a set input load *p*_1_, the pressure values *p*_2_, *p_c_*_1_ and *p_c_*_2_ can be calculated by the aforementioned Equations (1)–(4) and (7). Therefore, the acoustic amplification factor *K* of the SPAS can be confirmed by:

(8) $$K=\frac{p_{2}}{p_{1}}$$

In this way, the structural parameters of SPAS could be optimized by the mechanical amplification responsivity on basis of Equation (8).

## 3. Simulation on Acoustic Amplification Effect

### 3.1. Effect of SPAS Structural Parameters on Amplification Ratio

**Figure 3:**
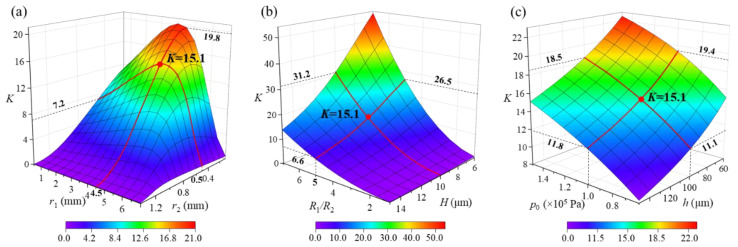
Simulation on (**a**) the effect of the radius *r*_1_ and *r*_2_ of the connecting rod on *K*, (**b**) the effect of the ratio of radius *R*_1_/*R*_2_ and the thickness *H* of two diaphragms, and (**c**) the effect of the height *h* and the initial pressure *p*_0_ of cavity 2 on *K*.

An investigation of the radius and thickness of diaphragm and rod as dominant structural parameters was introduced to evaluate the amplification ratio of SPAS. For simplified analysis, considering the reduced size and ease of fabrication for the SPAS, the material and thickness of the two diaphragms are identical by defining *H*_1_ = *H*_2_ = *H*. Taking a steel diaphragm as an example, *E*_1_ = *E*_2_ = *E* = 2.07 × 10^11^ Pa and *μ*_1_ = *μ*_2_ = *μ* = 0.29. The related simulation parameters for the design of SPAS are listed in Table 1. It should be added that since the fabrication of SPAS and the connection of the amplification structure with an F-P sensor were performed at room temperature and pressure, the initial pressure in cavity 2 was assumed to be about 1 × 10^5^ Pa in Table 1. Then, based on the established model mentioned above, Figure 3 shows the influence of structural parameters on *K.*

Referring to Figure 3a, although as a whole the increased *r*_1_ or reduced *r*_2_ contributes to an increase in *K*, the radius at both the upper and lower surfaces of the pressure conduction rod produces the non-monotonic effect on *K*. It can be seen from the red line in Figure 3 that for a certain *r*_2_ value (0.5 mm), *r*_1_ is confirmed as 4.5 mm instead of a maximum value to achieve an extreme value of 15.1 for *K*. In contrast, when *r*_1_ is chosen as 4.5 mm, *r*_2_ can be further reduced to obtain a higher *K*; however, a smaller *r*_2_ is limited by the fixation technology of the connecting rod and the supporting diaphragm at its lower endface. In other words, *r*_2_ followed by *r*_1_ is confirmed by the model simulation. In Figure 3b, *K* is directly positively proportional to *R*_1_/*R*_2_ and 1/*H*. Moreover, the enhancement effect of *R*_1_/*R*_2_ is more obvious than that of *H.* For example, for a specific diaphragm thickness of 10 μm, *K* equals to 1 when *R*_1_/*R*_2_ = 1; nevertheless, it correspondingly rises from 15.1 to 31.2 with an amplification factor of ~2.1 when *R*_1_/*R*_2_ changes from 5 to 7. Hence, the preference for dual thin diaphragms with a large area ratio should be given on the basis of the thinning process of diaphragm material and miniaturization of SPAS. In addition to the aforementioned main structural parameters, the reduced height (*h*) of cavity 2 and the increased initial pressure (*p*_0_) in the cavity also demonstrate clearly consistent effects on *K* as shown in Figure 3c. According to the red line in Figure 3c, for a cavity height of 100 μm at an approximate median point in the range of 60–150 μm, *K* is increased to 18.5 at 1.5 × 10^5^ Pa from 11.1 at 0.6 × 10^5^ Pa with a 66.7% rate of increase. By contrast, a 64.4% rate of increase for *K* is obtained when the cavity height (*h*) decreases from 150 μm to 60 μm at 1 × 10^5^ Pa. Although the magnitude of the impact with little difference, whether raising *p*_0_ or reducing *h* closely depends upon the sealing outer casing and miniaturization process of SPAS.

### 3.2. Effect of Diaphragm Parameters on Acoustic Amplification

**Figure 4:**
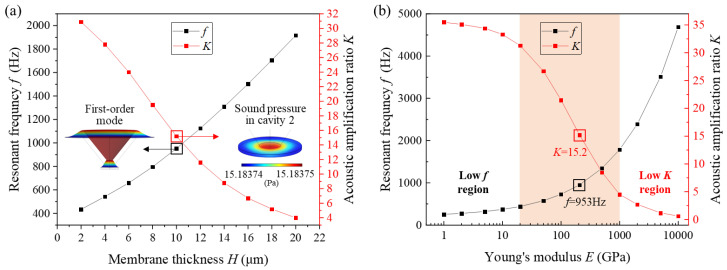
Acoustic simulation on (**a**) the effect of diaphragm thickness *H* on *K* and *f* and (**b**) the effect of Young’s modulus *E* of the diaphragm on *K* and *f*.

According to the above simulation on *K*, the structural parameters of dual diaphragms are major influence factors, wherein the diaphragm thickness is more critical regarding the limitation in the physical dimension of SPAS. Hence, acoustic amplification performance by COMSOL multiphysics simulation using the initial parameters in Table 1 mentioned above is performed. By imposing a 1-Pa dynamic acoustic pressure whose frequency ranges from 200 Hz to 10 kHz on diaphragm 1, the acoustic pressure response is investigated in cavity 2 to estimate the dynamic amplification performance. Assuming the excitation frequency is 20 Hz. Figure 4 illustrates the dynamic acoustic response (*f* and *K*) verse diaphragm thickness (*H*) and Young’s modulus (*E*) of the diaphragm, respectively, wherein *f* is the resonance frequency at the first vibration mode of the diaphragm. It can be clearly found in Figure 4a that *f* and *K* represent the opposite response to *H* or *E*. To achieve a wider frequency response for acoustic amplification, the thicker diaphragm is much better; however, the expense of lower *K* needs to be paid. Hence, a trade-off between *f* and *K* should be considered. It should be pointed out that the simulated sound pressures are, respectively, 15.18374 Pa and 15.18375 Pa at the edge and center of the diaphragm, with a negligible pressure difference of 10 μPa. That is to say, the acoustic pressure in cavity 2 can be regarded to be uniform. Similarly, in Figure 4b, the preferable response to *E* primarily lies in the region of 20–1000 GPa, as indicated in the orange area. In view of acoustic sensitivity evaluation at 1 kHz with a higher *K* value, the points closing to the intersection in Figure 4a are chosen so as to obtain *K* = 15.2 and *f* = 953 Hz, thereby confirming the corresponding film thickness of 10 μm and Young’s modulus of ~207 GPa. Note that the *K* value equals 15.2 by COMSOL simulation in Figure 4 agrees well with the one that is calculated as 15.1 in Figure 3, thus favorably signifying the availability of the established theoretical model for the SPAS.

## 4. Experiment and Result Analysis

### 4.1. SPSA Fabrication

**Figure 5:**
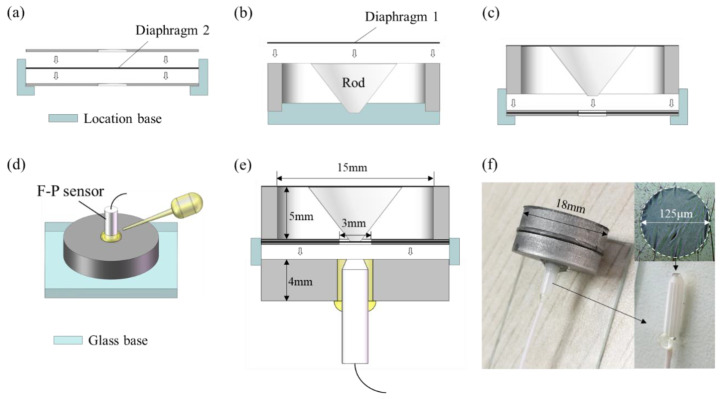
Fabrication process of the SPAS. (**a**) Clamping diaphragm 2 between two steel gaskets. (**b**) Bonding diaphragm 1 onto the endface of a steel casing. (**c**) Fixing the parts that make up the SPAS. (**d**) Inserting an F-P probe into a location base. (**e**) Assembling the casing with the locating base installed with an F-P probe by epoxy glue. (**f**) Picture of the assembled SPAS.

Figure 5 presents the fabrication process of the SPAS. As shown in Figure 5a, the diaphragm 2 was clamped between two 100 μm thickness steel gaskets with a 3 mm diameter inner hole by the use of epoxy glue. Then, as shown in Figure 5b, based on the same adhesive bonding method, the diaphragm 1 was glued onto the endface of a machined steel casing with an inner diameter of 15 mm and a height of 5 mm. Then, a PLA tapered rod with upper and lower diameters of 9 mm and 1 mm was 3D printed, whose upper surface was adhered to the diaphragm 1. After that, referring to Figure 5c, both the casing with the tapered rod and the gaskets holding the diaphragm 2 were glued together via a location base whose inner diameter is the same as the outer diameter of the casing. The following process is to insert an F-P probe into a steel location base that would be held together with the casing in the subsequent procedure. As indicated in Figure 5d, an F-P probe with a 13-layer graphene diaphragm with a thickness of ~4.3 nm was inserted into a location base with an inner diameter of 2.5 mm until it contacted the surface of a glass base. In this case, the epoxy glue was dipped into the joint between the F-P probe and the location plate in order to stabilize the two components. Next, in a similar manner, the steel plate with an F-P probe was rigidly attached to the steel casing by glue as given in Figure 5e. Note that the detailed process of transferring a graphene diaphragm onto the endface of a ferrule with an inner diameter of 125 μm in the inset in Figure 5f can refer to the Ref. [33], wherein a wetting transfer method was adopted. In short, firstly, the polymethyl methacrylate (PMMA) substrate was etched off by immersing the divided graphene sample into acetone solution for about 1 h. Secondly, the ferrule was moved down slowly toward the floating graphene diaphragm until it touched the graphene diaphragm. Finally, the graphene diaphragm-covered fiber-capillary tip assembly was then left to dry in a cabinet for about half an hour. In this way, the preparation of an F-P probe coated with the graphene diaphragm was completed. As a result, the SPAS assembled with the F-P probe was displayed in Figure 5f, wherein the SPAS shows the physical dimension of *Φ*18 mm × 9 mm. Additionally, the inset in Figure 5f shows that certain wrinkles occurred on the surface of the diaphragm, which was to a certain extent caused by the uneven stress in the diaphragm. The uneven film stress would affect the flatness of dynamic frequency response of the F-P acoustic sensor and the upper limiting frequency, which could be improved by optimizing a high-quality transfer method of graphene diaphragm in the future study. In addition, it should be added that during acoustic test, diaphragm 2 made of steel or thermoplastic polyurethanes (TPU) material was introduced successively to fabricate different SPAS devices for comparison of acoustic amplification behaviors, in combination with the use of reflective coating on diaphragm 2.

### 4.2. Acoustic Measurement Setup

**Figure 6:**
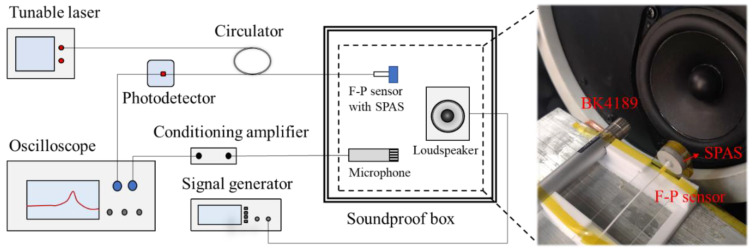
Experimental setup for the acoustic test.

To examine the sensitivity-enhancing effect induced by the introduction of SPAS, an acoustic pressure measurement setup was established as shown in Figure 6. A signal generator (DG5102, Rigol, Beijing, China) offered a dynamic acoustic signal in the range of 0.2–10 kHz via a conventional loudspeaker. The generated acoustic signal was detected simultaneously by the developed F-P sensor and a reference microphone (BK4189, BK, Nærum, Denmark) with a sensitivity of 50 mV/Pa, which were placed at the two symmetry positions closer to the loudspeaker along the central axis of the loudspeaker in a self-made soundproof box. The F-P sensor was driven by 1550 nm incident light with an optical power of −16.3 dB sent by a tunable laser (AP3350A, APEX, France) for the optimal voltage output. Then the resulting interference signal through a three-port circulator (6015-3-APC, Thorlabs, Newton, NJ, USA) was fed into an oscilloscope (DPO3054, Tektronix, Beaverton, OR, USA) for optical intensity demodulation through a low noise photodetector (PR-200K4177, Conquer, Beijing, China) with a 200 kHz bandwidth. In this way, the voltage output from BK4189 was amplified by a conditioning amplifier (BK1708, BK, Nærum, Denmark) and then external acoustic pressure applied on the diaphragm 1 could be determined by the amplified voltage and the reference microphone output. Hence, the sensitivity verse acoustic pressure can be obtained for the developed F-P sensor with or without SPAS. Then, the amplification ratio *K* in different acoustic frequencies can further be solved by the ratio of the two measured acoustic responses when the SPAS was used or not.

### 4.3. Results and Discussion

**Figure 7:**
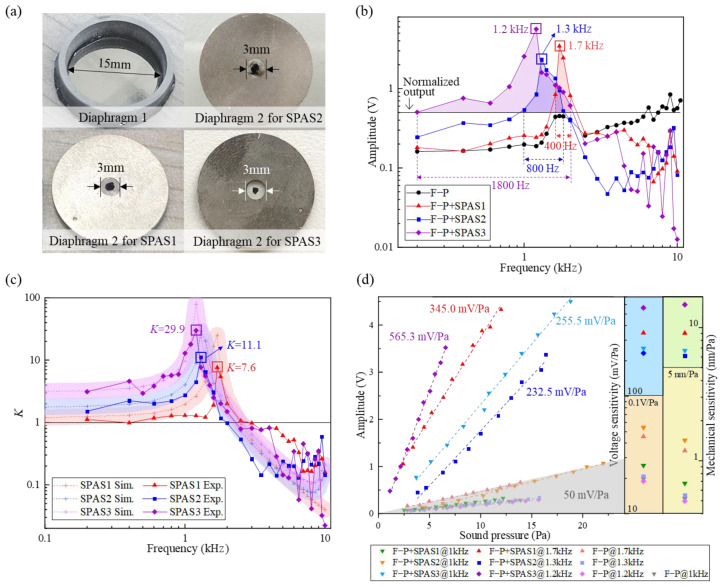
(**a**) Diaphragms 1 and 2 for the acoustic test. (**b**) Measured frequency response with or without SPAS, (**c**) *K* value and (**d**) acoustic sensitivity for the F-P sensors with SPAS1, SPAS2, and SPAS3. Note that the black horizontal line in (**b**) represents the normalized output from the reference microphone.

In order to optimize the SPAS for acoustic amplification, three types of SPASs, named as SPAS1, SPAS2, and SPAS3, were fabricated by employing a steel diaphragm, TPU diaphragm with nano-carbon power coating, and TPU diaphragm with black paint coating as diaphragm 2, respectively, as depicted in Figure 7a. Through the experimental setup in Figure 6, it can be concluded from the acoustic frequency response in Figure 7b that the F-P sensors with SPAS could remarkably achieve much higher output voltage; moreover, their responses near resonant frequencies are even superior to that (~500 mV) from the reference microphone. In addition, SPAS2 and SPAS3 can offer relatively stronger sound pressure signals than SPAS1, with the sacrifice of the slightly declining resonant frequency from 1.7 kHz to 1.3 kHz and then 1.2 kHz. It is further worth pointing out that in comparison with SPAS2, SPAS3 provided more preferable acoustic frequency output with a double peak amplitude in a wider band of 1.8 kHz, where the latter demonstrated a more sensitive response to sound information than BK4189. The corresponding *K* values were calculated by dividing the response from the F-P sensor using SPAS by the one without SPAS, as indicated in Figure 7c. Although the three types of SPASs obtained roughly identical bands corresponding to the region of *K* greater than 1 (0.2–2.5 kHz, 0.2–2 kHz and 0.2–2.3 kHz), which agrees well with the simulation data as labelled by dashed line with shadow area, SPAS3 exhibited the optimal amplification performance conforming to the sound-enhanced trend in Figure 7b due to higher *K* values in a comparatively broad frequency domain. The maximum *K* values corresponding to the resonant frequencies 1.7 kHz, 1.3 kHz, and 1.2 kHz for SPAS1, SPAS2, and SPAS3, respectively, were calculated as 7.6, 11.1, and 29.9. The varying *K* or amplitude response verse frequency is dependent upon the resonant amplification effect of the presented SPAS. Therefore, further research on a SPAS with a greater harmonic response with a wide and flat low-pass band is needed to improve the aforementioned frequency response fluctuations.

The acoustic sensitivities of these graphene-based F-P sensors with the SPAS under different acoustic pressure levels at the representative frequencies were measured as shown in Figure 7d. Compared with the F-P sensors without SPAS, those F-P probes with SPAS behaved more outstanding acoustic pressure characteristics. Take the SPAS3 among the three SPAS as an example. The F-P sensor with the SPAS3 exhibited the acoustic sensitivities of 255.5 mV/Pa @ 1 kHz and 565.3 mV/Pa @ 1.2 kHz, respectively. By comparison, the F-P sensor without SPAS only showed a sensitivity of ~20 mV/Pa. Hence, there is no doubt about the effective sound-enhanced effect induced by the SPAS. Additionally, although the enhanced sensitivity for the F-P sensor using the SPAS1 or SPAS2 was limited at typical 1 kHz, the voltage sensitivities of the F-P sensor were estimated to be 345.0 mV/Pa @1.7 kHz and 232.5 mV/Pa @1.3 kHz by using a least square fitting method with a fitting coefficient of 99.6% and 99.8%, respectively.

Note that the measured acoustic sensitivity mentioned above is the voltage sensitivity, which is also related to the signal conditioning unit in addition to the sensor performance itself. Thus, the mechanical sensitivity, closely concerned with the intrinsic response of the sensor, should also be evaluated on the basis of the mechanical response characteristic of the pressure-sensitive diaphragm. In this case, it can be inferred from the large deflection behaviors of circular graphene diaphragm [34] that the diaphragm deflection responses for the F-P sensor with the SPAS3 under acoustic pressure were derived as ~6.7 nm/Pa and ~14.8 nm/Pa, respectively, corresponding to 255.5 mV/Pa @1 kHz and 565.3 mV/Pa @1.2 kHz. The calculated mechanical sensitivity by far higher than 6 nm/Pa is obviously preceded over conventional diaphragm-type F-P acoustic sensors with the measured optimal sensitivity of ~2.38 nm/Pa in Ref. [34] and ~1.1 nm/Pa in Ref. [35]. Referring to Figure 7b, the F-P sensor with the SPAS3 also revealed a sensitivity-enhanced frequency band of 0.2–2.3 kHz (bandwidth: 2.1 kHz), which was obviously greater than the sound field enhancement bandwidth of ~1 kHz generated by a spiral-type acoustic tube in Ref. [20] and ~100 Hz confined by a single resonant peak excited by a Helmholtz resonator [22]. This phenomenon verified the advantage and the applicability of the presented external SPAS. Additionally, it further reveals that a higher resonant frequency as the cut-off frequency with a flat low-frequency band is oriented to structural improvement in future research.

## 5. Conclusions

In order to enhance the acoustic sensitivity for an F-P acoustic sensor via an amplification structure, a human ear-inspired cylindrical SPAS, whose outer diameter and height were 18 mm 9 mm, was designed by means of two circular diaphragms interconnected with a conical round rod. Then, the COMSOL-based sound field simulation of the amplification structure demonstrated the dominating influence of structural parameters of the diaphragm on the amplification ratio and resonant frequency response. According to the COMSOL simulation analysis, the radius ratio of the two circular diaphragms in SPAS was set as ~5, along with a radius ratio of 0.6 for the upper endfaces of the connecting round rod. Furthermore, steel and TPU materials were used in combination so as to fabricate three types of SPAS parts (SPAS1, SPAS2, and SPAS3). Subsequently, an F-P acoustic sensor with a multilayer graphene diaphragm suspended onto a ferrule with a diameter of 125 μm was inserted into the SPAS for acoustic test. The measured results showed that the SPAS3 achieved the optimal acoustic harmonic response that is higher than the reference microphone within the range of 0.2–2 kHz. Moreover, the maximum gain factor of ~29.9 for the SPAS3 was obtained at 1.2 kHz, which is obviously greater than 7.6 and 11.1 for the other two SPASs with a narrower bandwidth of 400–800 Hz. In this case, the enhanced acoustic sensitivity of 255.5 mV/Pa @ 1 kHz was achieved for the F-P sensor with the SPAS3, which is significantly superior to the conventional F-P acoustic sensor reported previously and the reference electric microphone (~50 mV/Pa @ 1 kHz).

## Figures and Tables

**Table 1:** Simulation parameters for the design of SPAS.

Structural Parameter	Value/mm	Material Parameter	Value
***R***_1_, ***R***_2_	7.5, 1.5	***E***	2.07 × 10^11^ Pa
***r***_1_, ***r***_2_	4.5, 0.5	***μ***	0.29
H	0.01	**Pressure in cavity 2**	**Value**
***h***	0.1	***p*_0_**	1.01 × 10^5^ Pa
